# Haplogroups of Mitochondrial DNA Differentiate Patterns of Cognitive Change Over 7 Years

**DOI:** 10.1093/geroni/igab046.1198

**Published:** 2021-12-17

**Authors:** Amber Watts, Elias Michaelis, Russell Swerdlow

**Affiliations:** 1 University of Kansas, Lawrence, Kansas, United States; 2 University of Kansas Alzheimer's Disease Center, Fairway, Kansas, United States

## Abstract

Mitochondrial DNA (mtDNA) may play an important role in Alzheimer’s disease (AD) and cognitive decline. A particular haplogroup of mtDNA (haplogroup J), has been observed more commonly in patients with AD than in cognitively normal controls. We used mtDNA haplogroups to predict change in cognitive performance over seven years. We hypothesized that haplogroup J would predict poorer cognitive function and steeper cognitive decline. We analyzed data from 140 cognitively normal older adults (age 65+) who participated in the University of Kansas Alzheimer’s Disease Center annual registry. We used factor analysis to create three composite scores (verbal memory, attention, executive function) from 11 individual cognitive tests. We performed latent growth curve modeling to describe trajectories of cognitive performance and change. We compared haplogroup H, the most common, to haplogroup J, the potential risk group. Results indicated haplogroup J carriers had significantly lower baseline performance (B=-.049, p< .01) and slower rates of improvement (B=-.046, p < .05) on tests of verbal memory compared to haplogroup H. For executive function, groups did not differ at baseline (B=.065, p>.10), but haplogroup J had slower rates of improvement (B=-.097, p < .01). There were no differences in attention across groups in performance (B=.135, p>.10) or change (B=-.01, p>.10). Our results reinforce the important role of mtDNA in changes to cognitive function with aging and imply that the effects of haplogroup J may vary across cognitive domains. Future research should investigate the mechanisms by which mtDNA might affect performance on specific cognitive domains across haplogroups.

